# The Unbearable Lightness of Health Science Reporting: A Week Examining Italian Print Media

**DOI:** 10.1371/journal.pone.0009829

**Published:** 2010-03-24

**Authors:** Luca Iaboli, Luana Caselli, Angelina Filice, Gianpaolo Russi, Eleonora Belletti

**Affiliations:** 1 Department of Emergency Medicine, Santa Maria Nuova Hospital, Reggio Emilia, Italy; 2 Department of Biomedical Sciences and Advanced Therapies, University of Ferrara, Ferrara, Italy; 3 Department of Nuclear Medicine, Santa Maria Nuova Hospital, Reggio Emilia, Italy; 4 Department of Transfusion Medicine, Santa Maria Nuova Hospital, Reggio Emilia, Italy; 5 Health Science Library, S. Anna Hospital, Ferrara, Italy; Mount Sinai School of Medicine, United States of America

## Abstract

**Background:**

Although being an important source of science news information to the public, print news media have often been criticized in their credibility. Health-related content of press media articles has been examined by many studies underlining that information about benefits, risks and costs are often incomplete or inadequate and financial conflicts of interest are rarely reported. However, these studies have focused their analysis on very selected science articles. The present research aimed at adopting a wider explorative approach, by analysing all types of health science information appearing on the Italian national press in one-week period. Moreover, we attempted to score the balance of the articles.

**Methodology/Principal Findings:**

We collected 146 health science communication articles defined as articles aiming at improving the reader's knowledge on health from a scientific perspective. Articles were evaluated by 3 independent physicians with respect to different divulgation parameters: benefits, costs, risks, sources of information, disclosure of financial conflicts of interest and balance. Balance was evaluated with regard to exaggerated or non correct claims. The selected articles appeared on 41 Italian national daily newspapers and 41 weekly magazines, representing 89% of national circulation copies: 97 articles (66%) covered common medical treatments or basic scientific research and 49 (34%) were about new medical treatments, procedures, tests or products. We found that only 6/49 (12%) articles on new treatments, procedures, tests or products mentioned costs or risks to patients. Moreover, benefits were always maximized and in 16/49 cases (33%) they were presented in relative rather than absolute terms. The majority of stories (133/146, 91%) did not report any financial conflict of interest. Among these, 15 were shown to underreport them (15/146, 9.5%), as we demonstrated that conflicts of interest did actually exist. Unbalanced articles were 27/146 (18%). Specifically, the probability of unbalanced reporting was significantly increased in stories about a new treatment, procedure, test or product (22/49, 45%), compared to stories covering common treatments or basic scientific research (5/97, 5%) (risk ratio, 8.72).

**Conclusions/Significance:**

Consistent with prior research on health science communication in other countries, we report undisclosed costs and risks, emphasized benefits, unrevealed financial conflicts of interest and exaggerated claims in Italian print media. In addition, we show that the risk for a story about a new medical approach to be unbalanced is almost 9 times higher with respect to stories about any other kind of health science-related topics. These findings raise again the fundamental issue whether popular media is detrimental rather than useful to public health.

## Introduction

Scientific journalism has a huge responsibility in improving our knowledge on health-related topics from a scientific perspective. Journalists have to translate and critically interpret risks and benefits of relevant scientific advances into an accurate, balanced, complete and understandable story for their readers. Though press news media are more likely to be trusted than advertisement newspapers [1], and have the potential to give the lay public more opportunities than ever before to become informed about health, until now the issue whether they do support or sabotage health is controversial [2]. Over the last 10 years it has been shown that information reported by popular media are detrimental to public health [3]. Most of the articles popularizing medical treatments provide inadequate or incomplete information about benefits, risks and costs of the specific treatments [4]–[7]. Conflicts of interest are rarely reported [8], [9], and existent financial ties between medical journalists and for-profit companies are hardly ever cited [10], [11].

These aspects have been mainly examined by studies focusing on the media coverage of selected target issues, such as the introduction of new potentially life-saving medicines [6], [7], [12] or the latest advances into a specific medical field published on the major biomedical journals [5], [13]. For example, Wilson et al. [12] analyzed UK national newspaper coverage of trastuzumab, a drug for early stage breast cancer treatment and Bubela et al. [5] examined the accuracy of media coverage of genetic research by reviewing the reporting of single-gene discoveries in major daily newspapers in Canada, US, UK and Australia. These studies have also recently inspired the creation of Web site projects in Australia (http://www.mediadoctor.org.au/), Canada (http://www.mediadoctor.ca/), UK (http://nhs.uk/news/Pages/Newsindex.aspx) and US (http://HealthNewsReview.org/) specifically aiming to adopt a monitoring approach over health news media coverage by the major national news organizations. In a recent work [4], HealthNewsReview.org project confirmed the poor quality of health news media covering stories about new medical treatments, tests, products and procedures.

The aim of the present study was twofold: on one hand, we asked whether similar journalistic shortcomings may be observable also when taking into account articles dealing with health-related topics other than the introduction of new prescription drugs or the description of the hottest medical breakthroughs. On the other hand, we attempted to adopt a wider explorative approach with respect to previous studies by evaluating health news reporting by all (Italian) national newspapers.

We addressed these issues by analyzing all kinds of health-related articles (i.e., articles aiming at improving the reader's knowledge about health from a scientific perspective, thus dealing with basic scientific research, as well as medical treatments, tests, products or procedures) appearing on the Italian newspapers over one sample week. The articles were evaluated by health-professional coders with respect to different divulgation parameters [4]. Moreover, we attempted to evaluate whether the analyzed articles were unbalanced (i.e., containing exaggerated or non correct claims about the discussed topic) or not. In particular, we were interested in assessing whether articles covering new treatments, tests, products and procedures were significantly less balanced than articles reporting on other topics.

## Methods

Through Nograziepagoio (http://www.nograziepagoio.it), an organization similar to US No Free Lunch, dealing with conflicts of interest in health, a group of volunteer health professionals was recruited to collect the Italian national daily newspapers and weekly magazines listed in the Italian ADS print certification [Accertamenti Diffusione Stampa, 14], published over a sample one-week period. Sport newspapers were excluded from collection. The week was selected randomly: daily newspapers were collected from the 25th to the 31st of May 2008, whereas weekly magazines were collected during the following week (1–7 June 2008). We chose subsequent weeks so that, given their different publishing time, daily newspapers and weekly magazines likely covered the same news.

Volunteers, that were blind to the aim of the study, were then asked to perform a first rough article selection, by collecting from each newspaper all stories dealing with any kind of health-related topic. If the newspaper or magazine under review had an enclosed supplement on health, the instruction was to select it all. Then, one researcher (LI) performed the final selection, by specifically picking out, among the articles gathered by the volunteers, those properly meeting with the definition of health science communication articles, i.e., articles supposedly aiming at improving the reader's knowledge on health-related topics from a scientific perspective. Hence, obituaries, book reviews, articles about health ethics, politics or economy, readers' letters, reports on injuries, juridical and police inquiries, announcements of future conferences, well-being/fitness articles and medical advertisements were all discarded. Identical health science communication articles reported in different newspapers were counted as one.

The selected articles were first evaluated according to size, i.e., the space occupied in relation to the dimension of the page in the newspaper. In addition, in order to measure the general space devoted to health science articles within daily newspapers and weekly magazines, the size of a given article (ranging from 0 in the case that no article was detected up to more than one page) was divided by the total number of pages in the newspaper publishing the article. Then, articles were analyzed independently by three health-professional coders (AF, GR, LI). These were physicians with different medical backgrounds (nuclear, transfusion and emergency medicine, respectively), asked to evaluate the content of each story through completion of a questionnaire addressing the following points: topic of the articles; costs, risks and benefits of articles covering new medical approaches, sources of information for the articles, disclosure of financial conflicts of interest and balance of the article.

Questionnaire and process of question coding are reported in table 1.

**Table 1:** Information about the questionnaire.

**Q1:** What is the main topic of the article?	Question to group articles according to the topic they dealt with. Coders had to tick one or multiple of the following options: basic scientific research, treatment, prevention, diagnosis, other.	k = 0.67
**Q2:** Is the story about a new treatment, procedure, test or product? If this is the case, are costs (a), risks (b) and benefits (c) of the new approach discussed? Are benefits reported in relative or absolute terms?	A story about a new medical treatment, procedure, test or product is incomplete if it does not address costs, potential risks and benefits. Benefits have to be described in absolute rather than relative terms. For example, a medical intervention reducing the incidence of myocardial infarction from 3.9% to 2.5% can be described as either being 34% (relative) or 1.4% (absolute) effective. Coders had to assess whether the story covered: costs of the approach and/or comparisons of these with alternative approaches; potential risks; absolute or relative benefits.	a: k = 0.88 b: k = 0.87 c: k = 0.79
**Q3:** Is the source of information for the topic discussed in the article quoted?	Journalists should always mention the source of information for the story they report in the article so that the readers can have access to it. Coders had to verify that the source of information was present and write it down (e.g., biomedical journals, congresses, interviewed expert opinion leaders, books,..).	k = 0.58
**Q4:** Is the source of information compared with existing alternative sources of information?	A story is expected to put the new approach being discussed into the context of existing alternatives. Coders were instructed to look for multiple sources of information mentioned in the article.	k = 0.73
**Q5:** Are financial conflicts of interest mentioned?	Journalists should be vigilant in disclosing relevant financial conflicts of interest of those they report about. Coders had to identify economical financial ties explicitly reported in the story.	k = 0.80
**Q6:** Is the article balanced or unbalanced?	Journalists should give a balanced description of the object topic of the article, by cautioning about interpreting study results or reporting information on new medical approaches. For example, unbalanced stories are those overestimating the benefits of a medical treatment showed in a single or uncontrolled study by not considering the limitations of such a study; or those incorrectly emphasizing the importance of a basic scientific discovery with claims that go far beyond the potential implications of the findings. Coders had to evaluate whether articles were balanced or unbalanced, i.e., containing exaggerate or incorrect claims either in the way the story is reported or the source of information describes the scientific results covered by the story.	k = 0.51

Third column indicates kappa Fleiss'es coefficient for inter-coder reliability computed after completion of the questionnaire by the three coders.

For some questions, additional procedures were performed in the following cases. Costs of new treatments, procedures, tests or products (see question Q2a in table 1) that were not mentioned by journalists, were checked online by consulting drugs databases available on the web (e.g., http://www.codifa.it). When the source of information (see questions Q3, Q4 in table 1) was a biomedical article, we collected further information, namely, whether the journal publishing the research paper was a peer-review one and we reported, if available, the journal impact factor [15], as an indicator of the quality of the source of information. Financial conflicts of interest (see question Q5 in table 1) that were not mentioned by journalists were systematically verified in the case the source of information for the story was a research article published on a biomedical journal or interviewed expert opinion leaders. In the former case, we reviewed the “Methods”, “Conflicts of Interest” and “Acknowledgements” sections of that article to determine whether the research was funded by the companies producing or supporting the drug or device that was the focus of the study. In the latter case, by Medline and Google Scholar tools we searched for studies published by the interviewed person in order to find out unreported financial conflicts of interest. Conversely, if the source of information was of another kind (e.g., congresses or representatives of patients' associations), it was not possible to find out undisclosed financial conflicts of interest.

### Statistical analysis

After completion of the questionnaire by the 3 coders, inter-coder reliability was assessed for each question by means of kappa Fleiss'es coefficient for multiple raters [16]. The measure calculates the degree of agreement with respect to that expected by chance and is scored as a number between 0 and 1. K values have been interpreted as according to Landis and Koch benchmark table [17].

For questions Q3 and Q6 the level of agreement among coders was lower (k = 0.58 and k = 0.51, respectively) compared to all other questions (k>0.6), thus discrepancies were directly discussed and solved by coders by also taking into consideration, when available, the retrieved original source of information for each story. Inter-coder agreement for each question is reported in table 1.

To explore whether articles covering new treatments, procedures, tests or products were significantly less balanced than articles reporting on other topics (Q6), we performed Chi-square test and computed the risk ratio between the probabilities to find unbalanced stories for the two types of health-related topics.

## Results

### Database

**Figure 1:**
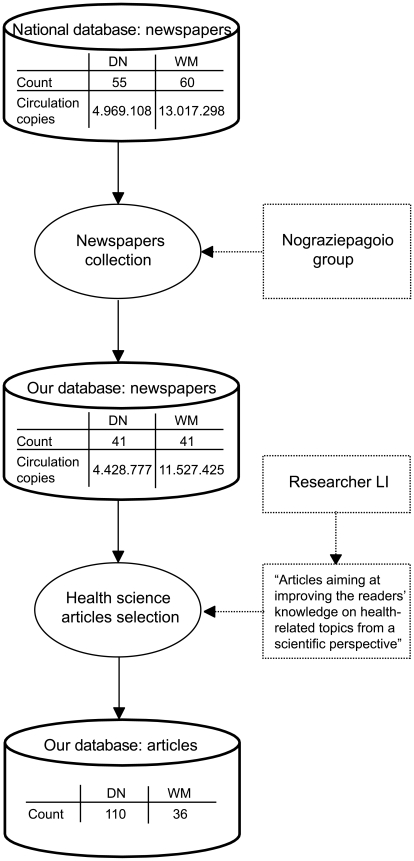
Newspapers collection and articles selection for the present database. Data on circulation copies are based on ADS database [14]. DN =  daily newspapers; WM =  weekly magazines.

Since sport newspapers were excluded from collection and some newspapers were not recovered, 41 out of the 55 national daily newspapers and 41 out of the 60 national weekly magazines (representing in both cases 89% of the total national circulation copies) were gathered by the collecting group. We identified in total 152 news stories, satisfying the criteria described in the Methods section. Since 6 of these were identical articles published on different inter-linked daily newspapers, they were counted as one. Therefore, the database for the present work included 146 articles: 110 coming from the daily newspapers and 36 taken from the weekly magazines (see Figure 1).

### Size of articles

The majority of the selected articles (51%) were less than one quarter of a page, 16% were from one to three pages, whereas the remaining articles were equally distributed across the other intermediate size categories listed in table 2. By looking at the size according to the type of newspapers, most of the stories selected from daily newspapers (61/110, 56%) were less than one quarter of page, whereas the majority of the stories from weekly magazines (15/36, 42%) were more than one page long.

**Table 2:** Size of articles.

	Article space in relation to page size					
	Small*	<0,25	0.25–0.5	0.5–1	>1	Total
**Daily Newspapers**	13 (12%)	48 (44%)	28 (25%)	13 (12%)	8 (7%)	110 (100%)
**Weekly Magazines**	1 (3%)	12 (33%)	4 (11%)	4 (11%)	15 (42%)	36 (100%)
**Sub-total**	14 (10%)	60 (41%)	32 (22%)	17 (11%)	23 (16%)	146 (100%)

*Small reports are of three paragraphs or less.

By averaging across newspapers the ratios obtained by diving the size of a given article by the total number of newspaper pages we found that, overall, Italian press dedicates 0.7% of the total content to health news.

### Main topics of the articles

Articles were mainly categorized as dealing with basic scientific research (40/146, 27%), medical treatments (36/146, 25%) or multiple topics among treatment, prevention and diagnosis (32/146, 22%). Percentages of articles assigned to the different topic categories provided in the first question of the survey are reported in table 3.

**Table 3:** Main topic of articles (Q1).

Topic	Daily newspapers *N = 110*	Weekly magazines *N = 36*
Basic scientific research	38 (35%)	2 (5%)
Treatment	27 (24%)	9 (25%)
Prevention	11 (10%)	13 (36%)
Diagnosis	3 (3%)	0 (0)
Multiple*	25 (23%)	7 (19%)
Other	6 (5%)	4 (12%)
Not classified	0 (0%)	1 (3%)

*Note that many articles were assigned to more than one topic among treatment, prevention and diagnosis.

By looking at the number of articles covering new medical approaches, 26 discussed a new treatment, 14 discussed a new procedure, 3 discussed a new test, and 6 discussed a new product, for a total of 49/146 (34%) discussing any new treatment, procedure, text or product. In contrast, 97 (66%) covered common medical treatments or basic scientific research.

### Costs, risks and benefits of new treatments, procedures, tests or products

Evaluation of costs, risks and benefits of the 49 stories covering new medical approaches revealed that costs were mentioned in 6 articles (12%). Interestingly, most of articles about new treatments (15/26) concerned costly drugs (<50 € in 2 cases, 50–500 € in 4 cases, 500–1000 € in 5 cases, >1000 € in 4 cases) already on the market but only 2 of these mentioned costs.

Risks were reported in 6/49 articles (12%).

A generic benefit was always (in 49/49 cases) reported in qualitative terms. However, benefits were never quantified as absolute values; they were expressed in relative terms in 16/49 cases (33%).

### Sources of information

In most of the articles (95%), the sources of information were clearly identifiable. They were of the following types: research articles published on biomedical journals (28%), interviewed expert opinion leaders (professors, researchers, head physicians, representatives of patients' associations or, less frequently, experts of companies) (59%), congresses (29%) or books (3%).

When the source of information was a research article published on a biomedical journal, we found that in most of the cases, the journal was a peer review one with an average IF of 15.3 (range: 1.5–52.6). Only in 9/146 stories (6%) more than one single source of information were cited.

### Disclosure of financial conflicts of interest

Financial conflicts of interest were explicitly reported in 13/146 stories (9%). Among the articles not reporting any financial conflict of interest (133/146, 91%), 15 were shown to underreport a conflict of interest that actually did exist (15/146, 9.5%), as we demonstrated by performing the additional procedures described in the *Methods*.

### Balance

Coders judged that articles were not balanced in 28/146 cases (19%). After discrepancies were solved by coders, according to procedures described in the *Methods* (inter-coder agreement for this question was k = 0.51), results did not change substantially: 27 out of 146 stories (18%) were judged unbalanced. The majority of the unbalanced stories were about new treatments, procedures, tests or products (22/49, 45%) and only 5 were stories reporting common treatments or basic scientific research (5/97, 5%) (Chi-square = 32.00, P<0.001), meaning that the relative risk for unbalanced stories between the two types of stories was 8.72 (CI: 8.28–9.16, P<0.05).

In the majority of the unbalanced stories, exaggerated or incorrect claims aimed at or had the effect to favor a new treatment, procedure, test or product.

### Case examples of unbalanced press stories

One story about a new treatment overestimated the results of a study testing a new anti-hypertensive drug. Whereas the original study concluded that there was no substantial difference between the new drug and one of the most commonly used anti-hypertensive drugs, the story only reported the efficacy of the new drug compared to placebo, thus incorrectly exaggerating its benefits on blood pressure. In a story covering a new procedure, it was incorrectly claimed that anti-obesity surgical operations might increase patients' survival up to 88%, suggesting that 1% of the Italian population would benefit from this procedure. Besides exaggerating benefits, this story also did not mention at all risks related to surgery. In one story about breast cancer, Positron Emission Tomography (PET) was incorrectly presented as a screening tool and a new genetic test, not yet validated, was described as essential to prevent relapses. Another story erroneously associated the fact that implantable digital defibrillators are more precise than canonical ones to the fact that they are more life-saving, thus increasing benefits of the new product though there is no effective advantage for health. A basic scientific research story reporting the results of a functional Magnetic Resonance Imaging study measuring brain activity of experimental participants asked to think about objects belonging to different semantic categories, exaggeratedly claimed (even in the title) that these findings will lay the ground for future mind reading.

## Discussion

Our analysis of the health-related coverage of Italian daily newspapers and weekly magazines shows that little attention, only 0.7% of the available printed space, is given to health-related issues. The quality of the information was also shown to be poor. For instance, the analyzed articles tended to ignore or minimize costs and risks. Moreover, though at least benefits were always mentioned in qualitative terms, they were quantified in relative rather than absolute terms in one third of the stories only. Identified sources of information were mainly research articles published on biomedical journals, communications from conferences or interviews to opinion leaders in a particular scientific field. Alternative sources of information were seldom taken into consideration. Our sample also showed that science journalism failed to disclose financial conflicts of interest in half cases. Importantly, a substantial portion of the examined stories were unbalanced. In particular, we observed that health news stories reporting on a new treatment, procedure, test or product, showed approximately a 9 times higher risk of unbalance than stories about common treatments or basic research.

These findings greatly overlap with those of previous studies about media coverage in English-speaking countries reporting under-disclosed important information by science journalism and raise again the fundamental issue whether popular media is detrimental rather than useful to public health. The very well known communication bias to minimize costs and risks and to express benefits in relative rather than absolute terms [4]–[7], [12] does not help neither physicians nor patients to be fully aware of costs [18] and potential harms on one side and to correctly perceive efficacy of treatments on the other [19]. Similarly to other works [8], [9], [20], we found that financial conflicts of interest were underreported. It is worthwhile noting that our results likely underestimate the extent of financial ties, since to disclose unaccounted conflicts of interest we could only rely on scientific literature, where such ties have been demonstrated to be underreported [21]–[25].

To our knowledge, few studies have examined the balance of media reporting so far. One of these [5] addressed the issue in stories covering genetic research articles and found that 11% of the analyzed stories had moderately to highly exaggerated claims. In contrast to this study, we explored the incidence of unbalanced stories in a sample of articles dealing with a wider range of health-related topics coming from different sources of information. Within this context, our finding that 18% of health science communication articles were unbalanced indicates that generally most of the Italian daily newspapers and weekly magazines stories accurately convey information about health-related topics. However, print media appear to overemphasize a particular class of topics concerning new treatments, procedures, tests and products. One possible explanation for this bias might be that journalists more or less inadvertently become one of the most effective vehicles for “selling” new medical approaches.

Our work has some limitations. First of all, we only considered print news media, though most of the health news information has been shown to come from television [26]. However, our choice was driven by the fact that newspapers typically contain more credible information than TV [27] and online news services [28] and allow individuals to engage in active, goal-directed searches for medical information [29]. Second, these findings are readable according to our construct of what constitutes good quality news. Our instruments were based on previous works and established guides [4], [5] but results may change with other assessment tools. Finally, coders had a specific scientific background that likely focused their attention on some particular aspects of the stories to the detriment of some others. Anyway, we were interested in analyzing print media coverage of health-related topics and we considered a key point that coders were health professionals.

Our study shows that print health science reporting, one of the major sources of news for clinicians and consumers in Italy has a number of problems that limit its reliability and make it “unbearably light”. Unreported costs and risks, emphasized benefits, undisclosed financial conflicts of interest and exaggerated claims about new medical approaches may create medicalization of non-diseases or incorrectly influence decisions about treatment choices and medical care [30]. As previously pointed out, newspapers do not exist to improve public understanding of health science, but have the potential to contribute to this [31], [32]. Among many examples of incomplete and unbalanced articles, some good reporting results about health can be found. However, this study suggests that there is much room for improvement.
